# Machine Learning for Investigation on Endocrine-Disrupting Chemicals with Gestational Age and Delivery Time in a Longitudinal Cohort

**DOI:** 10.34133/2021/9873135

**Published:** 2021-10-18

**Authors:** Hemi Luan, Hongzhi Zhao, Jiufeng Li, Yanqiu Zhou, Jing Fang, Hongxiu Liu, Yuanyuan Li, Wei Xia, Shunqing Xu, Zongwei Cai

**Affiliations:** ^1^School of Medicine, Academy for Advanced Interdisciplinary Studies, Southern University of Science and Technology, Shenzhen, China; ^2^State Key Laboratory of Environmental and Biological Analysis, Department of Chemistry, Hong Kong Baptist University, Hong Kong, SAR, China; ^3^Ministry of Education Key Laboratory of Pollution Processes and Environmental Criteria, College of Environmental Science and Engineering, Nankai University, Tianjin 300350, China; ^4^Key Laboratory of Environment and Health, Ministry of Education & Ministry of Environmental Protection, and State Key Laboratory of Environmental Health, School of Public Health, Tongji Medical College, Huazhong University of Science and Technology, Wuhan, Hubei 430030, China

## Abstract

Endocrine-disrupting chemicals (EDCs) are widespread environmental chemicals that are often considered as risk factors with weak activity on the hormone-dependent process of pregnancy. However, the adverse effects of EDCs in the body of pregnant women were underestimated. The interaction between dynamic concentration of EDCs and endogenous hormones (EHs) on gestational age and delivery time remains unclear. To define a temporal interaction between the EDCs and EHs during pregnancy, comprehensive, unbiased, and quantitative analyses of 33 EDCs and 14 EHs were performed for a longitudinal cohort with 2317 pregnant women. We developed a machine learning model with the dynamic concentration information of EDCs and EHs to predict gestational age with high accuracy in the longitudinal cohort of pregnant women. The optimal combination of EHs and EDCs can identify when labor occurs (time to delivery within two and four weeks, AUROC of 0.82). Our results revealed that the bisphenols and phthalates are more potent than partial EHs for gestational age or delivery time. This study represents the use of machine learning methods for quantitative analysis of pregnancy-related EDCs and EHs for understanding the EDCs' mixture effect on pregnancy with potential clinical utilities.

## 1. Introduction

Pregnancy is one of the most important periods for the mother and child. It involves a dynamic process associated with significant physiological changes and nutrient demands [1]. Small deviations from the norm during pregnancy result in complications in pregnancy including miscarriage and preterm birth [2, 3]. About 10-15% of pregnancy end in a miscarriage and around 10% end in preterm birth [4, 5]. Risk factors for miscarriage and preterm birth include advanced maternal age, immunological interactions, uterine anatomic abnormalities, hormonal imbalances, and environmental pollutions [5–7]. Exposure to environmental chemicals such as endocrine-disrupting chemicals (EDCs) has been implicated in pregnancy complications [8].

EDCs are commonly found in things you interact with every day such as food, waters, and plastic bottles. Exposure to EDCs such as bisphenol A, phthalates, has been suggested as a possible cause of pregnancy complications because of the harmful impact on reproductive and endocrine systems [8, 9]. Bisphenol A has been shown to affect endocrine functions by downregulating the expression of estrogen receptor, with the potential downstream effects of miscarriage and premature birth [10, 11]. Phthalates can also bind to the estrogen receptors and disturb sex steroid hormones, resulting in adverse pregnancy outcomes such as decreased length of gestation [12]. Besides the high concentration of bisphenol A and phthalates, exposures to the low concentration of parabens, benzotriazoles, and benzothiazoles were concerned. Recently published research found that the extremely low concentrations of parabens, benzotriazoles, and benzothiazoles in early pregnancy were also associated with the development of pregnancy complications such as gestational diabetes mellitus [13, 14]. While this is an important finding that low concentration of EDCs species may have a wide effect on pregnancy regulation through the endocrine systems, previous research did not focus on applying such quantitative knowledge in a machine learning fashion to reveal the interaction between EDCs and endogenous hormones (EHs) and identify the molecular underpinnings of pregnancy complications.

In the present study, we use liquid chromatography-mass spectrometry (LC-MS) to quantitate the concentration of 33 EDCs and 14 EHs with sampling of maternal urine at 1^st^, 2^nd^, and 3^rd^ trimesters from 2317 participants, investigating the interplay between EDCs and EHs throughout the pregnancy. We use a machine learning approach to integrate the EDCs and EHs to predict gestational age and find the potential candidate that affects the delivery time.

## 2. Results

### 2.1. Participant Characteristics

To capture the interaction between environmental pollutants and endogenous hormones during pregnancy that affects the gestational age, we established a large-scale pregnancy cohort and a design of urine sampling throughout different trimesters. A total of 2317 women with urine samples were assigned to discovery (*N* = 1986) and validation (test set, *N* = 331) cohorts. The demographic characteristics of the participants are shown in Table 1. The mean age of the pregnant women was 28 years at enrollment, ranging from 25 to 32 years. Among all newborns, 52.9% (*n* = 1051) were male and 47.1% (*n* = 935) were female in the discovery set; 53.2% (*n* = 176) were male and 46.8% (*n* = 155) were female in the test set.

### 2.2. Pregnancy Progression Associated with Environmental Pollutants

**Figure 1:**
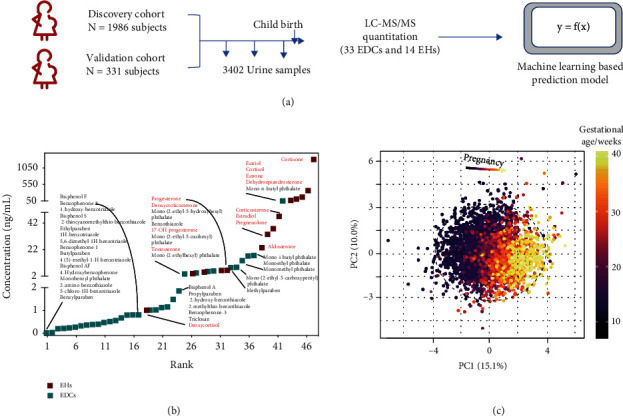
Study overview and EDC and EH characterization. (a) Overview of the study populations (cohorts) and schematic workflow. (b) The concentration distribution of 33 EDCs and 14 EHs in urine of pregnancy women. (c) Principal component analysis distributed individual samples according to pregnancy age. The two PCs explaining the largest part of the variation are shown.

We processed the 3402 samples from the 2317 subjects for the cohort (discovery and test set) and analyzed them according to our previous methods [15–18]. The concentrations of 47 compounds including 33 EDCs and 14 EHs were quantified by using a triple quadrupole mass spectrometer (Figure 1(a)). As shown in Figure 1(b), the average cortisone level was 1493 ng/mL, which has the highest concentration among the EDCs and EHs. The following EHs in order of decreasing concentration were estriol, cortisol, estrone, dehydroepiandrosterone, corticosterone, estradiol, pregnenolone, aldosterone, progesterone, deoxycorticosterone, 17-OH progesterone, and testosterone. Notably, the phthalate concentrations were ranged from 10.2 ng/mL to 119.8 ng/mL, which have the highest mean concentration among the EDCs. The average mono-n-butyl phthalate levels were 119.8 ng/mL, which was higher than many EHs, such as corticosterone, estradiol, pregnenolone, aldosterone, progesterone, deoxycorticosterone, 17-OH progesterone, and testosterone. Details of the concentration of 33 EDCs and 14 EHs are showed in Figure 1(b). Next, we analyzed the data globally with the PCA approach, in which all samples were distributed based on the first two principal components according to the gestational age (Figure 1(c) and Figure S1). The cumulative proportion values show that the first component explains 15.1% of the variability and the second explains an additional 10% for a cumulative total of 25.1%. These results suggest EDCs with extremely wide concentration ranges from 0.03 to 1493 ng/mL, worked together with EHs being associated with pregnancy progression or gestational age.

### 2.3. Gestational Age Linked to EDCs and EHs by Machine Learning

**Figure 2:**
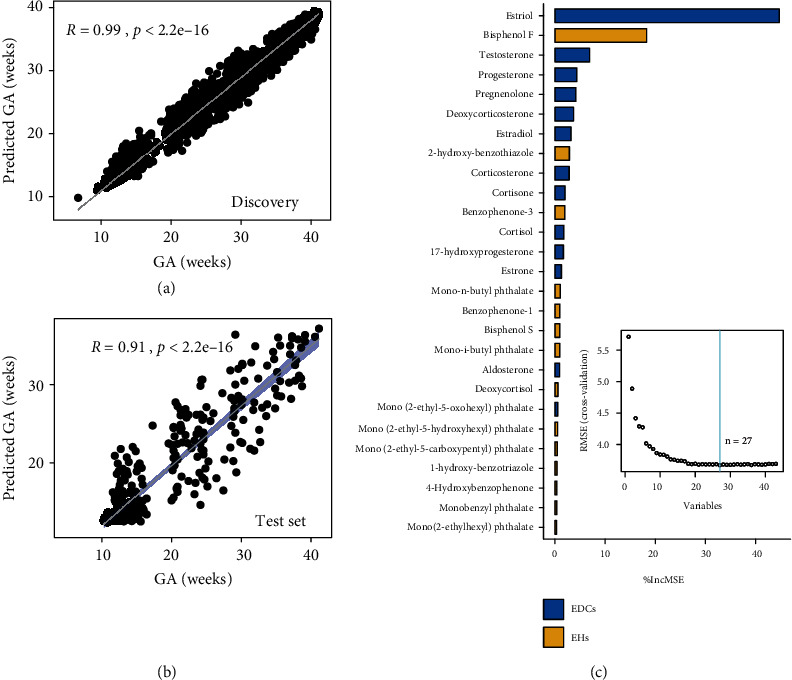
EDCs and EHs selected by recursive feature elimination (RFE) algorithm can accurately predict gestational age in both the discovery and validation cohorts. Gestational age predicted by machine learning model (predicted GA, *y*-axis) consisting of 27 compounds in the discovery (a) and the validation cohort (test set) (b). The 95% confidence interval for the linear regression is represented by the blue area. The percentage of increase in mean square error (%IncMSE) ranking the importance of 27 compounds that being selected by RFE algorithm (c).

We determined whether we can build a quantitative model based on the concentration of EDCs and EHs to predict gestational age for individual urine samples. We applied RFE-based feature selection with all 47 compounds to build the random forest model that shows optimal cross-validation performance for predicting gestational age in the discovery cohort (*N* = 3052 samples). We then ran the validation cohort data (test set, *N* = 350 samples) through the model established in the discovery cohort to measure the independent performance of the cross-validated model. In the cross-validation test of 3052 samples in the discovery cohort, the correlation between the predicted gestational age (predicted GA) in weeks and gestational age was calculated with a Pearson correlation coefficient (*R*) of 0.99 (*p* < 2.2*e* − 16) (Figure 2(a)). In the independent validation cohort, the model yielded a similar *R* of 0.91 (*p* < 2.2*e* − 16, test set) (Figure 2(b)). Twenty-seven of 47 compounds were selected by using RFE algorithm and combined to build the above predictive model, including 14 EDCs and 13 EHs. The percentage of increase in mean square error (%IncMSE) was used to rank the importance of 27 compounds influencing the prediction of gestational age (Figure 2(c)). Higher %IncMSE values indicate a more important predictor. As expected, estriol produced naturally by the placenta and fetus was the most important compound that influences the prediction of gestational age. Notably, bisphenol F, a popular substitute for bisphenol A in consumer products, is only less important than estriol that influences the prediction of gestational age (Figure 2(c)) but more important than bisphenol A. Bisphenol F has similar estrogenic and antiandrogenic effects on the mammalian endocrine system to those of bisphenol A. Furtherly, the least absolute shrinkage and selection operator (LASSO) was employed for feature selection for the 27 compounds in three trimesters (Table S3). Our findings highlight that there is no dose-dependent relationship between EDC levels and the compound importance (%IncMSE) in our predictive model. For example, the high concentration of phthalates with low %IncMSE values indicated a weak effect on the prediction of gestational age. Together, these results suggest EDCs and EHs can accurately predict the gestational age on the basis of urine samples from pregnant women.

### 2.4. EDCs and EHs Altered throughout Pregnancy

**Figure 3:**
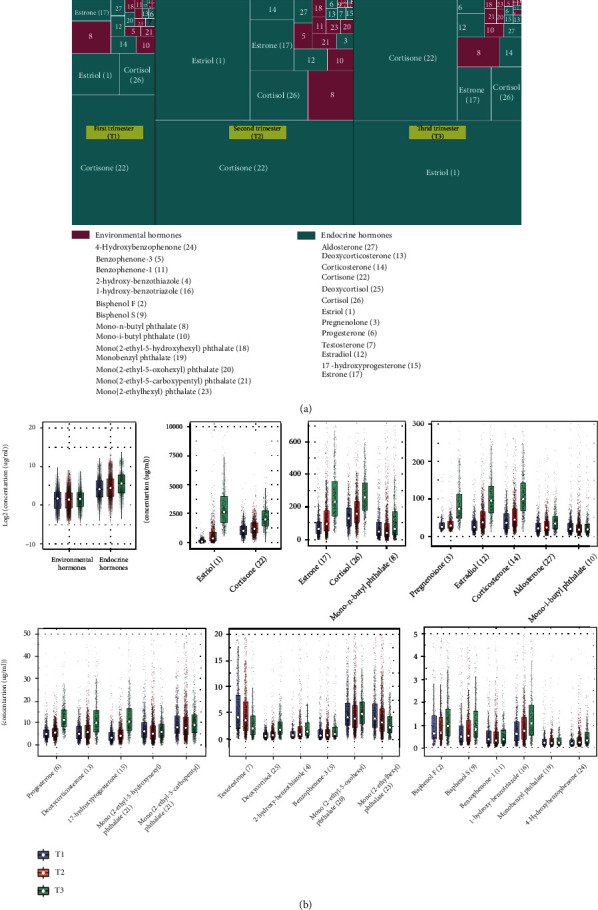
EDCs and EHs altered among different trimesters. (a) The “treeMap” displays the portion of EDCs and EHs for 27 compounds across three trimesters during pregnancy. Each rectangle's size is directly proportional to the compound concentration. (b) Box plots of EDCs and EHs among three trimesters. Asterisk (∗∗) denotes the significance with *p* < 0.05 and fold change > 1.2 or <0.8.

We performed a way of visualizing the hierarchical structure named “treeMap” to display the portion of EDCs and EHs for 27 compounds describe above across three trimesters during pregnancy. Each rectangle's size is directly proportional to the concentration of compounds. As shown in Figure 3(a), the highest total amount of those compounds was found in the second trimesters. The proportion of each EH was dramatically changed, while the proportion of each EDC was stable. For example, the proportion of cortisone (58.2%) has accounted for more than half of the total concentration at the first trimester and being deceased at the second trimester (46.9%) and the third trimester (32.9%). Reversely, the proportion of estriol (10.5%) was dramatically increased at the second trimester (25.4%) and the third trimester (46.8%) (Figure 3(a); Table S2). The proportion of each compound is shown in Table S2. Furtherly, we examined the changes of EHs throughout three trimesters. We observed an increase in total concentration of EHs. The concentration of each EH was significantly increased except that of testosterone throughout pregnancy (Figure 3(b)). We also examined the changes of EDCs throughout the three trimesters (Figure 3(b)). We found there were no changes in the total concentration of EDCs among three trimesters. Interestingly, the concentration of 4-hydroxybenzophenone was significantly increased in the second trimester when compared with the concentration of 4-hydroxybenzophenone at the first trimester, indicating the 4-hydroxybenzophenone may be regulated during the early pregnancy. During the later pregnancy, the concentrations of six EDCs including mono-n-butyl phthalate, 2-hydroxy-benzothiazole, bisphenol F, bisphenol S, 4-hydroxybenzophenone, and 1-hydroxy-benzotriazole were significantly increased at the third trimester compared to that of six EDCs in the second trimester. Notably, the concentration of mono(2-ethylhexyl) phthalate continued to decline throughout pregnancy and showed a significant fall in the third trimester (Figure 3(b)). Overall, among the 27 RFE-selected compounds in pregnancy, both EDCs and EHs were consistently altered throughout pregnancy, suggesting the intercorrelation to each other.

### 2.5. Association between EDCs and EHs during Pregnancy

**Figure 4:**
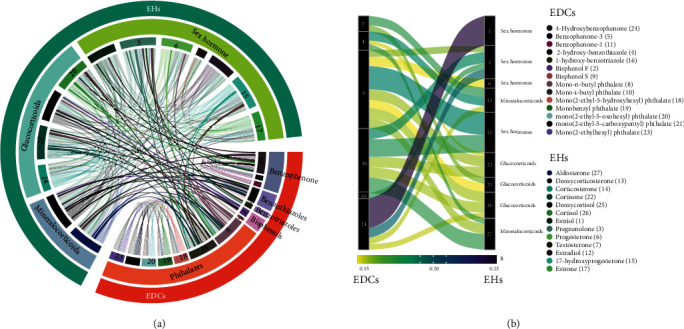
EDCs correlated with EHs during pregnancy. (a) A circular heat map showing the significant association between EDCs and EHs. Two compounds are connected if they are significantly correlated (adjusted *p* value < 0.05, *R* > 0.1). (b) An alluvial plot summarizing the strength or degree of relationship in the connected pairs. The weight of a line corresponds to its adjusted *p* value (scaled), and the color corresponds to its Spearman correlation coefficient (*R*).

To detect the association between EDCs and EHs that change during pregnancy, we performed correlation analysis on the 27 compounds mentioned above. Thirteen EHs of 27 compounds were divided into three functional groups according to their physiological function, e.g., sex hormones, glucocorticoids, and mineralocorticoids [19]. Fourteen EDCs of 27 compounds were divided into five groups according to their chemical structures, e.g., phthalates, bisphenols, benzotriazoles, benzothiazoles, and benzophenones. The association data between structure-based EDC groups and function-based EH groups were depicted in the form of a circular heat map (Figure 4(a)). Our results highlighted that the structure-based EDC groups were widely associated with function-based EHs, suggesting that each structure-based EDC group can affect the EH-related physiological functions. Six EDCs were significantly correlated with the function-based EH groups (adjusted *p* value < 0.001, *R* > 0.1). For example, 4-hydroxybenzophenone, mono-n-butyl phthalate, and mono-i-butyl phthalate were widely associated with sex hormone, mineralocorticoids, and glucocorticoids. Bisphenol F and 2-hydroxy-benzothiazole were associated with mineralocorticoids and glucocorticoids. Interestingly, 1-hydroxy-benzotriazole was specifically associated with sex hormones (Figure 4(b)).

### 2.6. Prediction for the Timing of Delivery with EDCs and EHs

**Figure 5:**
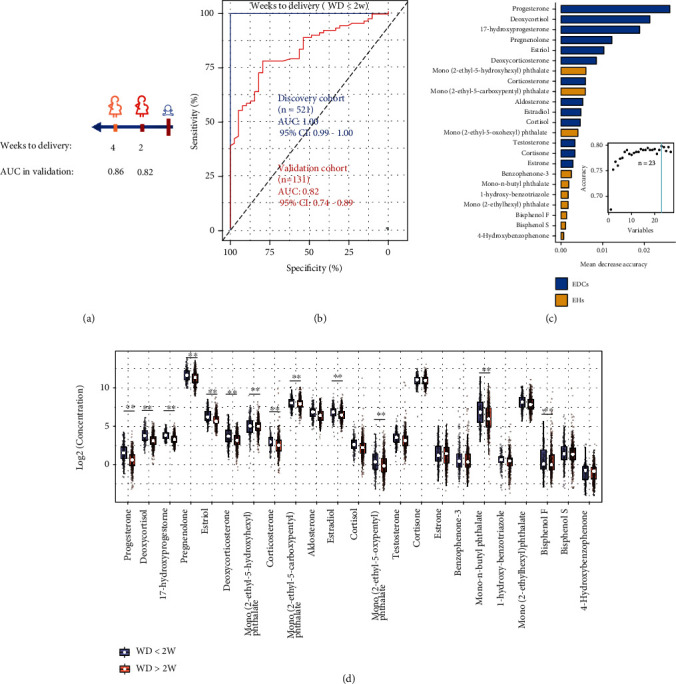
Altered EDCs and EHs linked with timing of delivery. (a) Summary of prediction models of 2 and 4 weeks approaching the delivery. The weeks to delivery were built using samples of the third trimester (>28 weeks). AUCs in the validation cohort (test set) are listed. (b) The machine learning model based on RFE-selected 23 compounds can accurately identify the third-trimester urine samples approaching the delivery (weeks to delivery [WD] < 2 w). (c) The mean decrease accuracy ranking the importance of 23 compounds that are being selected by RFE algorithm. (d) Box plots showing the concentration separations of EDCs and EHs before or after 2 weeks approaching the delivery. Asterisk (∗∗) denotes the significance with *p* < 0.05 and fold change > 1.2 or <0.8.

We then examined whether the EDCs and EHs can also predict the timing of a delivery event within a defined period (2 and 4 weeks from delivery) approaching the labor events (Figure 5(a)). The urine samples at the third trimester (*N* = 652 samples) were randomly divided into two independent cohorts. We applied RFE-based feature selection with the 27 compounds to build the random forest model that shows optimal cross-validation performance for predicting a delivery event in the discovery cohort. We then ran the validation cohort data through the model established in the discovery cohort to measure the independent performance of the built model. Firstly, we examined whether the EDCs and EHs can predict a delivery within 2 weeks (weeks to delivery [WD] < 2 w). The RFE-selected 23 compounds predicted an upcoming delivery event within 2 weeks in the discovery cohort with AUROC of 1.00 and validation cohorts with an AUROC of 0.82 (Figure 5(b) and Figure 5(c)). Similarly, the selected compounds can also be used to predict the timing of a delivery event within 4 weeks with AUROC of 0.86 (Figure 5(b)). The mean decrease accuracy was used to rank the importance of RFE-selected 23 compounds influencing the prediction of a delivery event (Figure 5(c)). We found that 8 EHs and 5 EDCs showed significantly increased concentration within 2 weeks approaching the delivery (Figure 4(d)). These results demonstrated that we can precisely categorize critical pregnancy stages in normal subjects by using the EDCs and EHs, and the EDCs play a key role in a delivery event during the pregnancy.

## 3. Discussion

In this study, we performed the LC-MS to accurately quantitate the concentration of 33 EDCs and 14 EHs with samplings of maternal urine from 2317 participants, investigating the interplay between EDCs and EHs throughout the pregnancy. We were able to quantify many of the EDCs revealed in previous studies (such as bisphenol A, bisphenol S, and phthalates) [17, 20], validating our approach. Those 33 quantified EDCs belong to emerging contaminants that began to be widely found in the aquatic environment and draw attention during vulnerable periods of pregnancy, while those 14 EHs are responsible for human reproduction and development. To our knowledge, comprehensive, unbiased, and quantitative analyses of 33 EDCs and 14 EHs associated with the timing of pregnancy in our study have not been reported. Also, we identified a wide variety of EDCs and EHs whose concentrations altered during pregnancy progression [13, 14, 17].

The alteration of EHs in an orchestrated manner during pregnancy maintains the maternal biological physiology during pregnancy and fetal growth. The estrogen hormones including estriol, estradiol, and estrone in pregnancy cause dramatic changes in the mother's vagina, cervix, uterus, and breast and significantly altered the nutrient metabolism [21], which was consisted with the constantly accumulated concentration of estrogen throughout the pregnancy (Figure 3(b)). Abnormal changes of estriol during the pregnancy were associated with an increased risk of preterm birth [22, 23]. Here, we revealed that the concentration of 4-hydroxybenzophenone was increased continuously and correlated with that of estriol. Also, we found estriol was the most important variable in our machine learning-based model for predicting gestational age (Figure 2(c)). Our results suggested the 4-hydroxybenzophenone may interfere with the physical process of estriol during pregnancy. Also, we detected a significantly increased concentration of progesterone and 17-hydroxyprogesterone in the third trimester. These two compounds can maintain the endometrium throughout pregnancy and are popularly employed to reduce the risk of miscarriage in pregnant women [24, 25]. Both progesterone and 17-hydroxyprogesterone were ranked as the top important variables in our machine learning-based model for predicting a delivery event (Figure 4(c)), validating our model. Interestingly, we observed the specific correlation between progesterone and phthalates. For example, progesterone was significantly correlated to mono-n-butyl phthalate, and 17-hydroxyprogesterone was significantly correlated to both mono-n-butyl phthalate and mono-i-butyl phthalate (Figure 4(b)), consistent with previous findings that phthalates have the potential to compete with the normal substrate binding of progesterone receptor [26] and interference progesterone secretion [27]. In addition, 5 phthalates out of 10 EDCs were ranked as the key variables in our machine learning-based model for predicting a delivery event, indicating the exposures of phthalates may be one of the high-risk factors for unexpected delivery [28, 29]. Our results highlighted that the role of specific bisphenols and phthalates is even more important than EHs for gestational age or delivery time. Comprehensive, unbiased, and quantitative analyses of EDCs and EHs, combining with the machine learning, enabled us to quantitatively examine the interplay between EDCs and EHs and understand the EDCs' effect on times of the progression of pregnancy. We found that using the EHs and EDCs, without any other inputs from clinical features, we can precisely determine the gestational age [30]. In addition, there was decreased accuracy of the predictive model when the EDCs were absent (AUROC = 0.81, Figures S2A–S2C). We also concerned that the triclosan and 4 parabens were excluded from our predictive model due to the relatively low importance scores for gestational age prediction, although the triclosan and parabens were suspected to potentially contribute to pregnancy complications [13, 31, 32].

To our knowledge, this is the first study to use machine learning methods for quantitative analysis of EDCs with delivery time. We found the EDCs and EHs can also be used to predict the timing of a delivery event within a defined period approaching the labor events, and the accuracy of this prediction method was similar to the previous reported literature (AUROC of 0.7-0.9) [30, 33]. In addition, our results can reflect the effect of environmental chemical exposure on human pregnancy. Emerging evidence indicates that the gestational age is associated with the EDCs (e.g., bisphenol A [34], phthalates [35], and parabens [36]), although the gestational age may be affected by many factors such as nutritional, medical, obstetric, and environmental factors [37]. EDCs are known to alter the EH concentration by interfering with the synthesis and distribution of EHs. For example, bisphenol A causes a decrease in the level of circulating testosterone in the human and rat [38]. EDCs have been shown to affect signal transduction in EH-responsive and EH receptor expression [10, 26]. Evidence also shows the ability of EDCs to cause increased inflammation and oxidative stress that affect gestational age [37]. However, our study has its limitations. The concentration of compounds in the urine may be affected by urine volume; the specific gravity (SG) of urine samples has been employed to reduce the urinary dilution effect [39]. Meanwhile, some individual EDCs interacting with the endocrine system were not included in this study, such as polybrominated diphenyl ethers (PBDE) [40], polychlorinated biphenyls [41], and perfluorooctane sulfonate (PFOS) [42]. In the future, we need to build models with more EDCs in our large-scale cohorts.

In summary, combining machine learning and the quantitative data of EDCs and EHs revealed the interaction of EDCs and EHs during pregnancy. We also demonstrated that the dynamic concentration information of EDCs and EHs can be used to predict gestational age with high accuracy in a cohort of pregnant women. There is a great need for quantitative evaluation of the EDCs' effect on EHs, as well as the timing of pregnancy: the vast majority of adults have evidence of exposure to EDCs that being the particularly significant risk of pregnancy complications to pregnant women. Our study demonstrated that the development of clinical methods with EDCs to time pregnancy is promising, which have the potential to benefit pregnant women and fetus developments.

## 4. Materials and Methods

### 4.1. Study Population

The study was approved by the Ethics Committee of the Tongji Medical College, Huazhong University of Science and Technology, and Wuhan Women and Children Medical Care Center with written information and informed consent obtained from all subjects. The participants were selected from a birth cohort and conducted between 2014 and 2015 at Wuhan Women and Children Medical Care Center in Wuhan, China. The recruitment criteria were described in our previous work [20]. In brief, pregnant women were invited to participate in the project at less than 16 weeks of gestation. Each pregnant woman was scheduled for three visits at different trimesters. According to the written consent, participants were free to drop out at any time. Finally, 2317 pregnant women were included in this study. Gestational weeks were estimated based on the last menstrual period and further confirmed by their first-trimester ultrasound examination. From each woman, overnight fasting urine was collected in a polypropylene container and then aliquoted and stored at −80°C. Characteristics of the subjects are shown in Table 1.

### 4.2. Sample Preparation and EDC and EH Measurements

Within discovery and test set cohorts, 3402 pregnancy urine samples from 2317 women were completely randomized and analyzed. The sample preparation methods and instrument used have already been reported in our previous reports [15–18]. Briefly, one milliliter of urine sample spiked with the isotope-labeled internal standard solution was treated with *β*-glucuronidase/sulfatase. After the incubation at 37°C overnight and hydrolysis, liquid-liquid extraction was employed for the extraction of compounds, and then, the supernatants were dried for storage and/or dissolved with acetonitrile/water for the instrumental analysis. The chromatographic separation of EDCs and EHs was achieved with C18 column, and elution system is listed in Table S4. Tandem mass spectrometry (MS/MS) data of EDCs and EHs were acquired using a Thermo Scientific™ TSQ Quantiva™ Triple Quadrupole mass spectrometer (Thermo Scientific, San Jose, CA). The multiple reaction monitoring (MRM) parameters were optimized and employed to quantify 33 EDCs and 14 EHs in samples (Table S1). The linearity of calibrations was obtained by plotting the peak area ratio (analyte/internal standard area responses) versus concentration.

### 4.3. Urinary Specific Gravity Measurements

A handheld digital refractometer (Atago Co. Ltd., Tokyo, Japan) at room temperature was employed to measure the specific gravity (SG) of urine samples for correcting the urinary dilution effect. The SG-adjusted concentrations were calculated using the following formula: *C*_sg_ = *C* × (SG_*m*_ − 1/SG − 1), where *C*_sg_ denotes the SG-adjusted concentration (ng/mL), *C* denotes the measured concentration, SG_*m*_ denotes the median SG in the population, and SG denotes the specific gravity values.

### 4.4. Computational Analyses

Software tools used for this study are available as open-source R packages (https://www.r-project.org, v4.0.2). For key analyses, these include “ropls” and “statTarget” for multivariate statistical analysis [43], “circlize” and “ggalluvial” for EDC and EH correlations [44], “randomForest” and “caret” for training and plotting classification and regression models, and “treemap” and “ggplot2” for data visualization.

We applied principal component analysis (PCA) to examine the overall distribution of the sample data (with all 47 compounds in 3402 samples) according to the gestational age (by the ropls package). The Pareto scaling was used for data pretreatment before PCA. The correlations were examined between 33 EDCs and 14 EHs. We performed the “corr.test” function in the “psych” R package to calculate spearman correlation coefficients. All *p* values were adjusted for multiple testing using the false discovery rate (FDR).

Random forest is a supervised machine learning algorithm for both classification and regression. The use of this method minimizes the risk of overfitting, and the method is relatively robust against noise and outliers. The recursive feature elimination (RFE) function in the caret R package for feature selection was furtherly employed to determine the minimal number of top compounds with the lowest root mean square error (RMSE) for regression or highest accuracy for classification. We applied RFE with 10-fold cross-validation in the discovery dataset to select compounds to build the random forest model to predict gestational age. The building model was applied to the validation cohort for prediction and verification. A linear fitting from the above evaluations was performed between the predicted value and the actual values to assess the performance of the predictive model.

For samples collected at third trimester (>28 weeks), a similar discovery and validation workflow were employed to build a random forest model with an RFE algorithm for predicting the categorical labels of delivery within 2 or 4 weeks. To estimate the confidence interval for the area under receiver operating characteristic curve (AUROC), we used the “pROC” package to perform bootstrapping 10000 times and calculate the 95% confidence interval for AUROC. The mean decrease accuracy was used to evaluate the importance of compounds in the built random forest classifier.

## Figures and Tables

**Table 1:** Demographics and birth characteristics of the discovery and validation cohorts.

	Discovery (*N* = 1986)	Test set (*N* = 331)
Demographics		
Maternal age at birth (years)	28.6 ± 3.3	28.8 ± 3.3
Weight (kilograms)	54.0 ± 7.7	55.0 ± 8.5
Height (centimeters)	161.0 ± 4.4	161.29 ± 4.4
BMI	20.8 ± 2.6	21.1 ± 3.1
Birth characteristics		
Gestational age (weeks)	39.3 ± 1.2	39.3 ± 1.4
Birth weight (grams)	3306 ± 406	3327 ± 453
Birth length (centimeters)	50.2 ± 1.5	50.2 ± 1.6
Gender of child, no. (%)		
Male	1051 (52.9)	176 (53.2)
Female	935 (47.1)	155 (46.8)

Values are presented as the means (SDs) or numbers (percentages).

## Data Availability

All data needed in the paper are present in the paper and in the supplementary section. Additional data which are related to this paper may be provided upon request.

## Supplementary Materials

Supplementary Materials Table S1: the list of 33 EDCs and 14 EHs. Table S2: the portion of EDCs and EHs for 27 compounds across three trimesters during pregnancy. Table S3: variables with coefficient values from LASSO model for three trimesters. Table S4: the LC elution system for EDCs and EHs. Figure S1: the loading plot of principal component analysis. Figure S2: EHs were employed to predict gestational age in both the discovery and validation cohorts.
