# Supplementary material for: Machine Learning for Investigation on Endocrine-Disrupting Chemicals with Gestational Age and Delivery Time in a Longitudinal Cohort
Source: Research (Wash D C). 2021 Oct 18;2021:9873135. doi: 10.34133/2021/9873135 (PMC8548981; doi:10.34133/2021/9873135)
Supplement: Supplementary Materials — Table S1: the list of 33 EDCs and 14 EHs. Table S2: the portion of EDCs and EHs for 27 compounds across three trimesters during pregnancy. Table S3: variables with coefficient values from LASSO model for three trimesters. Table S4: the LC elution system for EDCs and EHs. Figure S1: the loading plot of principal component analysis. Figure S2: EHs were employed to predict gestational age in both the discovery and validation cohorts. [file 9873135.f1.docx]

**Supplementary Materials**

**Titles**

**Machine Learning for Investigation on Endocrine-Disrupting Chemicals with Gestational Age and Delivery Time in A Longitudinal Cohort**

Machine Learning for EDCs on Gestational Age and Delivery Time

**Authors**

Hemi Luan^1,2^, Hongzhi Zhao^2,3^, Jiufeng Li^2^, Yanqiu Zhou^2^, Jing Fang^2^, Hongxiu Liu^4^, Yuanyuan Li^4^, Wei Xia^4^, Shunqing Xu^4*^, Zongwei Cai^2*^

**Affiliations**

1. School of Medicine, Academy for Advanced Interdisciplinary Studies, Southern University of Science and Technology, Shenzhen, China

2. State Key Laboratory of Environmental and Biological Analysis, Department of Chemistry, Hong Kong Baptist University, Hong Kong, SAR, China

3. Ministry of Education Key Laboratory of Pollution Processes and Environmental Criteria, College of Environmental Science and Engineering, Nankai University, Tianjin, 300350, China

4. Key Laboratory of Environment and Health, Ministry of Education & Ministry of Environmental Protection, and State Key Laboratory of Environmental Health, School of Public Health, Tongji Medical College, Huazhong University of Science and Technology, Wuhan, Hubei 430030, China

* To whom correspondence may be addressed

Name and address for correspondence:

Prof. Zongwei Cai; E-mail: [zwcai@hkbu.edu.hk](mailto:zwcai@hkbu.edu.hk); Tel.: +852-34117070; fax: 34117348;

Prof. Shunqing Xu; E-mail: [xust@hust.edu.cn](mailto:xust@hust.edu.cn); Tel: 86-27-83657705; fax: 86-27-83657781;

| **Table S1 The list of 33 EDCs and 14 EHs** | | |  |  |  |
| --- | --- | --- | --- | --- | --- |
| ***Class*** | ***Subgroup*** | **Full** | **Mass transitions**  **(Q1-Q3)** | **Polarity** | **Internal standards** |
| *EDCs* | Bisphenols | Bisphenol S | 249-108 | Negative | Bisphenol S-13C12 |
| *EDCs* | Bisphenols | Bisphenol A | 227-212 | Negative | Bisphenol A-13C12 |
| *EDCs* | Bisphenols | Bisphenol AF | 335-265 | Negative | Bisphenol A-13C12 |
| *EDCs* | Triclosan | Triclosan | 287-35 | Negative | Triclosan-13C12 |
| *EDCs* | Bisphenols | Bisphenol F | 199-105 | Negative | Bisphenol A-13C12 |
| *EDCs* | Benzotriazoles | 1H-benzotriazole | 120-65 | Positive | 1-hydroxy-benzotriazole-D4 |
| *EDCs* | Benzotriazoles | 1-hydroxy-benzotriazole | 136-64 | Positive | 1-hydroxy-benzotriazole-D4 |
| *EDCs* | Benzotriazoles | 5,6-dimethyl-1H-benzotriaole | 13477 | Positive | 1-hydroxy-benzotriazole-D4 |
| *EDCs* | Benzotriazoles | 4(5)-methyl-1-H-benzotriazole | 134-77 | Positive | 1-hydroxy-benzotriazole-D4 |
| *EDCs* | Benzothiazoles | Benzothiazole | 136-65 | Positive | 1-hydroxy-benzotriazole-D4 |
| *EDCs* | Benzothiazoles | 2-hydroxy-benzothiazole | 152-80 | Positive | 1-hydroxy-benzotriazole-D4 |
| *EDCs* | Benzothiazoles | 2-methylthio-benzothiazole | 182-167 | Positive | 1-hydroxy-benzotriazole-D4 |
| *EDCs* | Benzothiazoles | 2-amino-benzothiazole | 151-109 | Positive | 1-hydroxy-benzotriazole-D4 |
| *EDCs* | Benzotriazoles | 5-chloro-1H-benzotriazole | 154-99 | Positive | 1-hydroxy-benzotriazole-D4 |
| *EDCs* | Benzotriazoles | 2-thiocyanomethylthio-benzothiazole | 239-221 | Positive | 1-hydroxy-benzotriazole-D4 |
| *EDCs* | Parabens | Methylparaben | 151-92 | Negative | Methylparaben-ring-13C6 |
| *EDCs* | Parabens | Ethylparaben | 165-92 | Negative | Ethylparaben-ring-13C6 |
| *EDCs* | Parabens | Propylparaben | 179-92 | Negative | n-propylparaben-ring-13C6 |
| *EDCs* | Parabens | Butylparaben | 193-92 | Negative | n-butylparaben-  ring-13C6 |
| *EDCs* | Parabens | Benzylparaben | 227-92 | Negative | n-butylparaben-  ring-13C6 |
| *EDCs* | Phthalates | Monomethyl phthalate | 179-77 | Negative | Monoethyl phthalate-D4 |
| *EDCs* | Phthalates | Monoethyl phthalate | 193-77 | Negative | Monoethyl phthalate-D4 |
| *EDCs* | Phthalates | Mono(2-ethyl-5-carboxypentyl) phthalate | 307-159 | Negative | Mono(2-ethyl-5-carboxypentyl) phthalate-13C4 |
| *EDCs* | Phthalates | mono(2-ethyl-5-hydroxyhexyl) phthalate | 293-145 | Negative | mono(2-ethyl-5-hydroxyhexyl) phthalate-D4 |
| *EDCs* | Phthalates | mono(2-ethyl-5-oxohexyl) phthalate | 291-143 | Negative | mono(2-ethyl-5-oxohexyl) phthalate-13C4 |
| *EDCs* | Phthalates | Mono-i-butyl phthalate | 221-77 | Negative | Mono-n-butyl phthalate-D4 |
| *EDCs* | Phthalates | Mono-n-butyl phthalate | 221-77 | Negative | Mono-n-butyl phthalate-D4 |
| *EDCs* | Phthalates | Monobenzyl phthalate | 255-183 | Negative | Monobenzyl phthalate-D4 |
| *EDCs* | Phthalates | mono(2-ethylhexyl) phthalate | 277-134 | Negative | mono(2-ethylhexyl) phthalate-D4 |
| *EDCs* | Benzophenones | 4-Hydroxybenzophenone |  | Negative | Benzophenone-3-13C12 |
| *EDCs* | Benzophenones | Benzophenone-1 | 213-91 | Negative | Benzophenone-3-13C12 |
| *EDCs* | Benzophenones | Benzophenone-8 | 243-123 | Negative | Benzophenone-3-13C12 |
| *EDCs* | Benzophenones | Benzophenone-3 | 227-211 | Negative | Benzophenone-3-13C12 |
| *EHs* | Glucocorticoids | Cortisone | 361-163 | Positive | Cortisone‐d7 |
| *EHs* | Glucocorticoids | Cortisol | 363-121 | Positive | Cortisol‐d4 |
| *EHs* | Glucocorticoids | Deoxycortisol | 347-109 | Positive | Corticosterone‐d8 |
| *EHs* | Mineralocorticoids | Aldosterone | 361-315 | Positive | Cortisol‐d4 |
| *EHs* | Glucocorticoids | Corticosterone | 347-121 | Positive | Corticosterone‐d8 |
| *EHs* | Mineralocorticoids | Deoxycorticosterone | 331-97 | Positive | Corticosterone‐d8 |
| *EHs* | Sex hormones | Progesterone | 315-97 | Positive | Progesterone‐d9 |
| *EHs* | Sex hormones | 17-OH progesterone | 331-97 | Positive | Corticosterone‐d8 |
| *EHs* | Sex hormones | Pregnenolone | 317-159 | Positive | Progesterone‐d9 |
| *EHs* | Sex hormones | Estrone | 271-159 | Positive | Cortisone‐d7 |
| *EHs* | Sex hormones | Estradiol | 255-159 | Positive | Cortisol‐d4 |
| *EHs* | Sex hormones | Estriol | 271-159 | Positive | Cortisone‐d7 |
| *EHs* | Sex hormones | Testosterone | 289-97 | Positive | Corticosterone‐d8 |
| *EHs* | Sex hormones | Dehydroepiandrosterone | 271-253 | Positive | Cortisol‐d4 |

| **Table S2 the portion of EDCs and EHs for 27 compounds across three trimester during pregnancy** | | | | |
| --- | --- | --- | --- | --- |
| **Compounds** | **Class** | **1^st^ trimester** | **2^nd^ trimester** | **3^rd^ trimester** |
| *Aldosterone* | EHs | 1.2 | 0.97 | 0.63 |
| *Benzophenone-1* | EDCs | 0.35 | 0.52 | 0.04 |
| *Benzophenone-3* | EDCs | 0.82 | 1.05 | 0.17 |
| *Bisphenol F* | EDCs | 0.04 | 0.05 | 0.04 |
| *Bisphenol S* | EDCs | 0.15 | 0.08 | 0.11 |
| *Corticosterone* | EHs | 2.32 | 1.97 | 1.74 |
| *Cortisol* | EHs | 7.82 | 6.33 | 4.2 |
| *Cortisone* | EHs | 58.16 | 46.9 | 32.87 |
| *Deoxycorticosterone* | EHs | 0.32 | 0.27 | 0.2 |
| *Deoxycortisol* | EHs | 0.05 | 0.04 | 0.04 |
| *Estradiol* | EHs | 1.51 | 1.64 | 1.68 |
| *Estriol* | EHs | 10.47 | 25.44 | 46.82 |
| *Estrone* | EHs | 4.38 | 4.94 | 4.74 |
| *Monobenzyl phthalate* | EDCs | 0.01 | 0.01 | 0 |
| *Mono(2-ethyl-5-carboxypentyl) phthalate* | EDCs | 0.74 | 0.79 | 0.46 |
| *Mono(2-ethyl-5-hydroxyhexyl) phthalate* | EDCs | 0.65 | 0.51 | 0.29 |
| *Mono(2-ethylhexyl) phthalate* | EDCs | 0.45 | 0.47 | 0.2 |
| *Mono(2-ethyl-5-oxohexyl) phthalate* | EDCs | 0.39 | 0.42 | 0.27 |
| *Mono-i-butyl phthalate* | EDCs | 1.69 | 1.28 | 0.68 |
| *Mono-n-butyl phthalate* | EDCs | 6.72 | 4.99 | 3.29 |
| *1-hydroxy-benzotriazole* | EDCs | 0.07 | 0.05 | 0.02 |
| *2-hydroxy-benzothiazole* | EDCs | 0.04 | 0.06 | 0.03 |
| *4-Hydroxybenzophenone* | EDCs | 0.01 | 0.01 | 0.01 |
| *17-hydroxyprogesterone* | EHs | 0.24 | 0.19 | 0.2 |
| *Pregnenolone* | EHs | 0.69 | 0.56 | 1.04 |
| *Progesterone* | EHs | 0.3 | 0.24 | 0.19 |
| *Testosterone* | EHs | 0.4 | 0.22 | 0.06 |
| * the portion of 27 compounds across three trimesters during pregnancy is directly proportional to the compounds concentration. | | | | |

**Table S3 Variables with coefficient values from LASSO model for three trimesters**

|  | | | |
| --- | --- | --- | --- |
| ***Compounds*** | **1^st^ trimester** | **2^nd^ trimester** | **3^rd^ trimester** |
| *Estriol* | 0 | 0.00197129 | 0.00017251 |
| *Bisphenol F* | 0 | 0.14213702 | 0.00047516 |
| *Pregnenolone* | 0 | 0 | 0.01372902 |
| *2-hydroxy-benzothiazole* | 0 | 0.09222817 | 0.08738398 |
| *Benzophenone-3* | 0 | -1.72E-05 | 0.00094714 |
| *Progesterone* | 0 | -0.0509829 | 0.10134966 |
| *Testosterone* | 0 | -0.1131436 | -0.0280951 |
| *Mono-n-butyl phthalate* | 0 | 0 | 0 |
| *Bisphenol S* | 0 | 0 | 0 |
| *Mono-i-butyl phthalate* | 0 | 0 | -0.0009178 |
| *Benzophenone-1* | 0 | 0 | 0.00241162 |
| *Estradiol* | 0 | 0 | 0 |
| *Deoxycorticosterone* | 0 | -0.0246674 | 0 |
| *Corticosterone* | 0 | -0.0021186 | 0 |
| *17-hydroxyprogesterone* | 0 | 0 | 0.05214102 |
| *1-hydroxy-benzotriazole* | 0 | 0 | 0 |
| *Estrone* | 0 | 0.00077737 | 0 |
| *Mono(2-ethyl-5-hydroxyhexyl) phthalate* | 0 | 0 | 0 |
| *Monobenzyl phthalate* | 0 | -0.0109907 | 0 |
| *Mono(2-ethyl-5-oxohexyl) phthalate* | 0 | 0 | 0 |
| *Mono(2-ethyl-5-carboxypentyl) phthalate* | 0 | 0 | 0 |
| *Cortisone* | 0 | -0.0002663 | 0 |
| *Mono(2-ethylhexyl) phthalate* | 0 | 0 | 0 |
| *4-Hydroxybenzophenone* | 0 | 0.62074406 | -0.035887 |
| *Deoxycortisol* | 0 | -0.0118443 | 0.10274946 |
| *Cortisol* | 0 | 0 | -0.0034913 |
| *Aldosterone* | 0 | -0.0134996 | 0 |

| ***Table S4 the LC elution system for EDCs and EHs.*** | | | | |
| --- | --- | --- | --- | --- |
| *METHODS* | Column | Gradient | Mobile Phase | Compounds |
| *1* | Acquity BEH C18 column (100 mm*2.1 mm, 1.7 μm, Waters) | 0 min, 30% B; 9 min, 52% B; 10.5–12 min, 95% B; 12.5–15 min, 30% B. | water (A) and ACN (B) | EHs |
| *2* | Thermo Scientific Betasil C18 column (2.1 mm*100 mm, 3 mm) | 1.5 min, 25% B; 11.5 min, 80% B; 12 min, 100% B; 12.2 min, 25% B; 15min, 25% B | water (A)  and acetonitrile (B). | Bisphenols, Benzophenones, Parabens, Triclosan |
| *3* | Waters UPLC C18 column (2.1mm*100 mm, 1.7 μm) | 5 min, 25% B; 7.5 min, 60% B; 8-10 min, 100% B; 10.5 min, 25% B; 13min, 25% B | Water with 0.1% formic acid (A) and acetonitrile (B). | Phthalates |
|  | Thermo Hypersil GOLD (1.9 μm, 100 × 2.1 mm; Thermo) | 0.5 min, 10% B; 9.5 min, 83% B; 9.5-12.5 min, 100% B; 12.5-13min, 10% B | Water with 0.1% formic acid (A) and acetonitrile (B). | Benzotriazoles |

**Figure S1.** The loading plot of principal component analysis


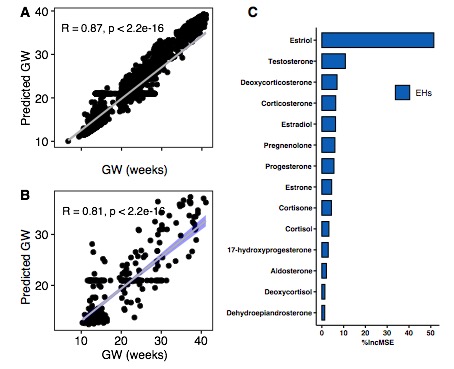


**Figure S2.** EHs were employed to predict gestational age in both the discovery and validation cohorts. Gestational age predicted by machine leaning model (Predicted GA, y axis) consisting of 14 EHs in the Discovery (A) and the validation cohort (test set) (B). The 95% confidence interval for the linear regression is represented by the blue area. The percentage of increase in mean square error (%IncMSE) ranking the importance of 14 EHs (C).
